# The In Vitro Immunomodulatory Effects of Gold Nanocomplex on THP-1-Derived Macrophages

**DOI:** 10.1155/2022/6031776

**Published:** 2022-02-10

**Authors:** Duaa Abuarqoub, Nouf N. Mahmoud, Rand Zaza, Rana Abu-Dahab, Enam A. Khalil, Dima A. Sabbah

**Affiliations:** ^1^Department of Pharmacology and Biomedical Sciences, Faculty of Pharmacy and Medical Sciences, University of Petra, Amman 11196, Jordan; ^2^Cell Therapy Center, The University of Jordan, Amman 11942, Jordan; ^3^Department of Pharmacy, Faculty of Pharmacy, Al-Zaytoonah University of Jordan, Amman 11733, Jordan; ^4^School of Pharmacy, The University of Jordan, Amman 11942, Jordan

## Abstract

**Introduction:**

This study is aimed at investigating the immunological response after treating THP-1 cells with gold nanorods conjugated with a phosphatidylinositol 3-kinase (PI3K*α*) inhibitor. *Methodology.* Gold nanorods were synthesized and functionalized with cholesterol-PEG-SH moiety, and the treatment groups were as follows: nanocomplex (a drug-conjugated gold nanorods), free drug (phosphatidylinositol 3-kinase (PI3K*α*) inhibitor), and GNR (the nanocarrier; cholesterol-coated gold nanorods). THP-1 cells were differentiated into macrophages and characterized by measuring the expression of macrophage surface markers by flow cytometry. Then, differentiated cells were activated by lipopolysaccharide (LPS). Afterwards, activated macrophages were treated with the different treatments: nanocomplex, free drug, and GNR, for 24 hrs. After treatment, the production of the inflammatory cytokines measured at gene and protein levels by using qPCR and CBA array beads by flow cytometry.

**Results:**

Our results show that THP-1 cells were successfully differentiated into macrophages. For inflammatory cytokine expression response, nanocomplex and free drug showed the same expression level of cytokines at gene level, as the expression of IL-1*β*, IL-6, and TNF-*α* was significantly downregulated (*p* < 0.0005, *p* < 0.0005, *p* < 0.00005), respectively, while IL-8, IL-10, and TGF-*β* were all upregulated in a significant manner for nanocomplex (*p* < 0.00005, *p* < 0.00005, *p* < 0.00005) and free drug treatment group (*p* < 0.00005, *p* < 0.05, *p* < 0.05) compared to the control untreated group. While in the GNR group, IL-6 and TNF-*α* were downregulated (*p* < 0.005, *p* < 0.00005), and IL-12p40 (*p* < 0.00005) was upregulated all in a statistically significant manner. While at protein level, cells were treated with our nanocomplex: IL-1*β*, IL-6, TNF-*α*, and IL-12p70 and were significantly decreased (*p* < 0.00005,*p* < 0.005,*p* < 0.05,*p* < 0.00005), and IL-10 was found to be significantly increased in culture compared to the untreated control group (*p* < 0.005). For free drug; IL-1*β* and IL-12p70 were significantly decreased (*p* < 0.00005, *p* < 0.00005), while a significant increase in the secretion levels of IL-10 only was noticed compared to the untreated group (*p* < 0.005). For GNR treatment groups, IL-1*β*, TNF-*α*, and IL-12p70 were significantly decreased (*p* < 0.00005, *p* < 0.05, *p* < 0.00005).

**Conclusion:**

We can conclude that our nanocomplex is a potent effector that prevents tumoral progression by activating three main immunological strategies: switching the surface expression profile of the activated macrophages into a proinflammatory M1-like phenotype, downregulating the expression of proinflammatory cytokines, and upregulating the expression level of anti-inflammatory cytokines.

## 1. Introduction

The utilization of nanoparticles as drug delivery nanosystems is considered one of nanotechnology's most crucial biomedical applications [1, 2]. Surface conjugation or uploading of small molecules into the nanoparticles could substantially enhance the drugs' solubility, stability, and pharmacokinetic properties [3–5]. In addition, conjugation of drugs to nanoparticles could improve their targeting potential and cellular internalization [6, 7]. Gold nanoparticles have several applications in medicine due to their unique properties related to their ease of synthesis and surface functionalization, tracking within the biological systems and their unique plasmonic properties [8, 9]. PI3K pathway is involved in cancer development, progression, and development of resistance towards chemotherapy [10]. In our previous work, we have conjugated our new promising phosphatidylinositol 3-kinase (PI3K*α*) inhibitor to gold nanoparticles where they demonstrated enhanced cytotoxicity against MCF-7 breast cancer cell line [11]; moreover, our recent work showed that the conjugated nanoparticles system has modulated the expression of PI3K*α* at both gene and protein levels in comparison to the drug or the gold nanorods separately [12]. However, the application of these new synthesized compounds in medicinal industry must be based on understanding all the biological properties of these compounds and being aware of all their toxicological effects, in addition to investigating their impact on different biological pathways that are involved in the characterization and functionalization of the cell [13, 14].

Nanoparticles can interact with the immune system cells in several aspects [15]; activation of the immune responses was observed upon exposure to different types of nanoparticles. Although these nanoparticles target specific cells, their interactions with other body cells cannot be avoided or neglected [16]. From this point of view, the study of the interaction between nanoparticles and other immune cells is a necessity to determine the nature of the immunological response that will result from their use in therapy. The introduction of such nanoparticles into the body can result in their recognition by immune cells which translates into an immune response that can lead to a serious medical problem [16, 17]. Additionally, the changes in immune cell phenotype upon interaction with the nanoparticle could be harnessed and employed in therapeutic approaches for immunological diseases, as it was shown previously that the alteration in the immune cell phenotype after treating cells with biological modifiers or a nanoparticles will induce the apoptosis and necrosis cell death modality of the treated cells ([18]). Herein, we aimed to investigate the immunological response that will result in vitro after using the nanocomplex (a drug-conjugated gold nanorods), the free drug (phosphatidylinositol 3-kinase (PI3K*α*) inhibitor), and GNR: cholesterol-coated gold nanorods, in addition to the control untreated group. The presented data indicate the interaction between macrophages and the investigated particles and compounds and a first step on evaluating the clinical implications of using these particles in therapeutic settings.

## 2. Materials and Methods

### 2.1. Synthesis and Characterization of Gold Nanorods and Their Functionalization with the Drug (Phosphatidylinositol 3-Kinase (PI3K*α*) Inhibitor)

Gold nanorods were synthesized and functionalized with the Cholesterol-PEG-SH moiety (Nanosoft Polymers, USA), and the obtained nanorods coated with cholesterol were conjugated with the drug as described previously by our group [11]. The obtained drug-conjugated gold nanorods were characterized by optical absorption, surface charge, particle size, and infrared spectra as described previously [11, 19].

### 2.2. Cell Culture of THP-1

The human monocytic cell line THP-1 (ATCC, USA) was maintained in RPMI 1640 media (Hyclone, USA) supplemented with 2 mM L glutamine (Gibco, USA), 100 U/ml of penicillin streptomycin (Gibco, USA), and 20% fetal bovine serum (Gibco, USA) in addition to 4.5 g/L glucose (Sigma, USA) and cultured in ultra-low attachment plates (Corning, USA). Cells were cultured on seeding density 1 × 10^5^ cells/ml. Media was exchanged every 2-3 days.

### 2.3. Differentiation into Macrophages

To induce the differentiation potential of THP-1 monocytes toward macrophages, THP-1 cells were seeded in tissue culture plates as the following: for 6 well plate, 2 × 10^5^ cells/ml and 5 ml were added to each well.

For 24 well plate, 1 ml was added/well. After 24 hrs of seeding, cells were treated with 100 nM phorbol 12-myristate 13-acetate (PMA) for 24 hrs. After the incubation period, nonadherent cells were removed by aspirating them. Cell's adherence is an indicator of successful differentiation into macrophages.

### 2.4. Characterization: Monocytes vs. Macrophages

To compare the variation in the expression profile of THP1 before and after differentiation into macrophages, 1 × 10^6^ cells/ml were harvested and stained with the following markers: CD11b-PE (ebioscience, USA), CD68-FITC (ebioscience, USA), CD14-PE-cy7 (BD, Biosciences, USA), CD206-PE (BD, Biosciences, USA), HLA-DR-PerCPCy5.5(BD, Biosciences, USA), and CD45-FITC (BD, Biosciences, USA). Samples were acquired and analyzed with FACS DIVA software Version 8 on a FACS Canto II flow cytometer (BD, Biosciences, USA) and Flowlogic 7.3 software.

### 2.5. Activation of Macrophages with LPS

To evaluate the drug/carrier-macrophage interaction; the differentiated macrophages were stimulated by using lipopolysaccharide 1 *μ*g/ml (LPS, Santa Cruz) for 24 hrs following that, medium was replaced with fresh media containing the following drugs nanocomplex (a drug-conjugated gold nanorods) 2 nM, the free drug (phosphatidylinositol 3-kinase (PI3K*α*) inhibitor) 50 mg/ml, and GNR: cholesterol-coated gold nanorods 2 nM for further 24 hrs and compared to the control untreated cells. After the incubation period, media were collected from treated cells and stored at-80°C.

### 2.6. Surface Receptor Expression after Drug Treatment

To evaluate whether our treatments, nanocomplex (a drug-conjugated gold nanorods), the free drug (phosphatidylinositol 3-kinase (PI3K*α*) inhibitor), and GNR: cholesterol-coated gold nanorods can do phenotype alteration by changing the expression of surface immune receptors or can modulate the intercellular communication; the surface marker expression was evaluated accordingly by flow cytometry.

First, 1 × 10^6^ cells/ml were harvested and collected by using 0.25%Trypsin EDTA (Gibco, USA). Then, cells were washed with PBS and centrifuged at 300 x g for 5 min. After that, cells were resuspended in stain buffer (BD, USA) and stained with the following flourscinated antibodies: CD11b-PE (ebioscience, USA), CD68-FITC (ebioscience, USA), CD14-PE-cy7 (BD, Biosciences, USA), CD206-PE (BD, Biosciences, USA), HLA-DR-PerCPCy5.5 (BD, Biosciences, USA), and CD45-FITC (BD, Biosciences, USA), in dark for 30 min at room temperature. Next, samples were washed with cell wash (BD. USA) and centrifuged at 300 x g for 5 min, followed by a resuspension step with 300 *μ*l of PBS. Samples were acquired and analyzed with FACS DIVA software Version 8 on a FACS Canto II flowcytometer (BD, Biosciences, USA).

### 2.7. Quantification of Inflammatory Cytokine's Release

#### 2.7.1. Multiplex Method-Cytometric Bead Array (CBA)

The impact of the nanocomplex (a drug-conjugated gold nanorods), the free drug (phosphatidylinositol 3-kinase (PI3K*α*) inhibitor), and GNR (cholesterol-coated gold nanorods) on THP-1-derived macrophages was evaluated by detecting the expression level of selected cytokines by using human inflammatory cytokines CBA array beads (BD, Biosciences, USA) by flow cytometry.

Supernatants were thawed and analyzed with the multiplex cytokines' detection systems CBA array human inflammatory cytokines (BD, Biosciences, USA). All samples were prepared and analyzed according to the manufacturer's instructions. All samples were acquired and analyzed on a FACS Canto II (BD, Biosciences, USA) and Flowlogic 7.3 software.

#### 2.7.2. Gene Expression (qPCR)

To further look into how treating cells with the nanocomplex, the free drug, and GNR would affect gene expression, qPCR was performed.

Treated cells were harvested and collected by using 0.25%trypsin EDTA (Gibco, USA). Then, RNA was extracted. RNA was extracted from treated cells and the control cells by using TRIzol hybrid method (Qiagen, USA).

One *μ*g of total RNA was used to synthesize cDNA by using Go script (Promega, USA). Q-PCR analyses were performed with SYBR Green PCR master mix reagent (Takara, USA) using CFX96 thermal cycler (Bio-Rad, USA). The PCR conditions were as follows: denaturation 95°C for 10 s, annealing 58°C for 15 s, and extension 72°C for 10 s of each PCR cycle and repeated for 35 cycles. The relative amount or fold change of the target gene was normalized relative to the level of human housekeeping gene cyclophilin gene (PPIA) and the differentiated cells stimulated with PMA before treatment. The specific primer set for analysis is listed in Table 1.

## 3. Results

### 3.1. Differentiation of THP-1 Cells into Macrophages

To confirm the success of differentiation from monocytes into macrophages, surface marker expression profile was evaluated by flow cytometry.

**Figure 1:**
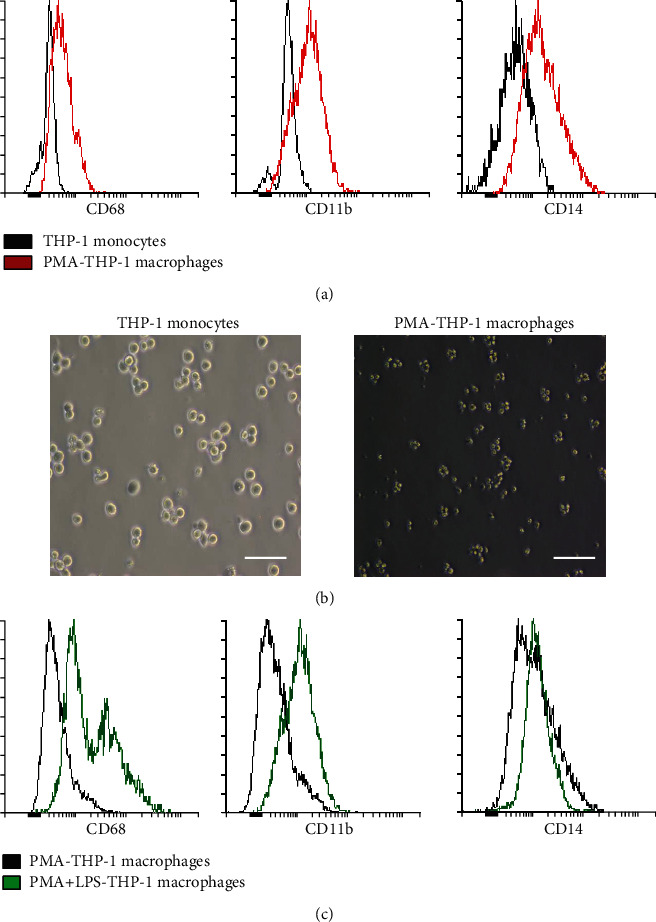
Characterization of THP-1 cells. (a) Flow cytometric histograms of THP-1 monocytes and PMA-THP-1 treated cells for 24 hrs. (b) The morphological appearance of THP-1 cell line cultured in RPMI media: THP-1 monocytes and macrophages; PMA treated THP-1. (c) Flow cytometric histograms of PMA-THP-1 macrophages and PMA+LPS-THP1 macrophages activated by LPS for 24 hrs.

THP-1 monocytic cells were differentiated into macrophages by treating cells with PMA. The expression profile of differentiated macrophages was distinguished from THP-1 monocytic cells. The expression of the following markers: CD68 and CD11b was highly upregulated in the differentiated macrophages compared to THP1-monocytes (Figure 1(a)).

Additionally, the morphological appearance of macrophages was distinguished from THP-1 monocytic cells, as macrophages are known by their adherence to the tissue culture plates (Figure 1(b)).

Differentiated macrophage cells were activated by using LPS. Remarkably, the CD68 was upregulated after the activation of macrophages by LPS, and the expression of CD68 was 50%. Moreover, For CD11b, the activated cells showed the same upregulation pattern as CD68, as the expression percentage was 80% (Figure 1(c)).

### 3.2. Surface Receptor Expression in the Presence of Treatments (Polarization of Macrophages)

**Figure 2:**
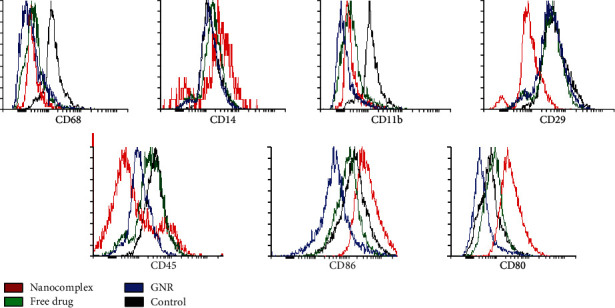
Flow cytometric histograms of PMA+LPS-macrophages surface expression markers after treatment with: nanocomplex (a drug-conjugated gold nanorods), the free drug (phosphatidylinositol 3-kinase (PI3K*α*) inhibitor), and GNR; cholesterol-coated gold nanorods for 24 hrs, compared to the control untreated cells.

After treating differentiated cells with different compounds: nanocomplex (a drug-conjugated gold nanorods), the free drug (phosphatidylinositol 3-kinase (PI3K*α*) inhibitor), and GNR (cholesterol-coated gold nanorods), the expression profile of the obtained macrophages was discrete. Cell surface markers: CD14, CD80, and CD86 were upregulated when cells were treated with nanocomplex, compared to the other treated groups and untreated group, while CD45, CD68, and CD29 were downregulated compared to the control untreated group. Moreover, all treated groups showed a down regulation of CD11b and CD68 compared to the control untreated group. Interestingly, the nanocomplex treatment appears to drive the polarization of these cells into a proinflammatory macrophage phenotype by increasing the expression of CD86, CD80, and CD14. For free drug treatment, no difference was observed for the expression profile of these markers, except for CD11b and CD68. Regarding the nanocarrier GNR, a downregulation in the expression of the following markers was observed: CD45, CD80, CD86, CD68, and CD11b (Figure 2).

### 3.3. Cytokine Expression in THP-1-Derived Macrophages after LPS Stimulation

To further study the effects of our compounds on macrophages, gene expression profiles and cytokine concentrations in culture media supernatants were evaluated for each macrophage treatment group.

#### 3.3.1. Gene Expression Level

**Figure 3:**
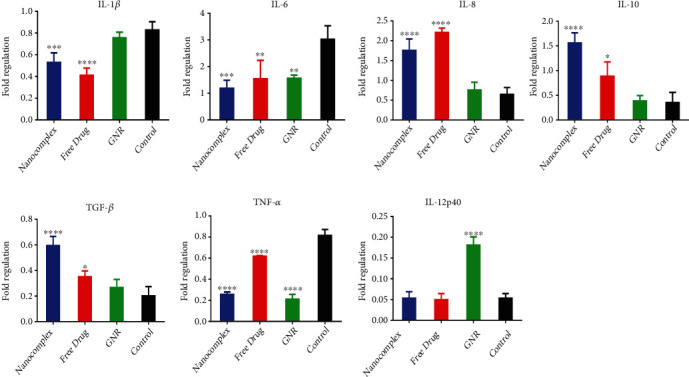
Measurement of the gene expression level of cytokines secreted by macrophages treated with the following: nanocomplex (a drug-conjugated gold nanorods), the free drug (phosphatidylinositol 3-kinase (PI3K*α*) inhibitor) and GNR; cholesterol-coated gold nanorods, and compared to the control untreated cells by qPCR.

As for the expression of cytokines at gene level, in the nanocomplex treatment group, the gene expression for IL-1*β*, IL-6, and TNF-*α* was all downregulated upon treatment with nanocomplex (*p* < 0.0005, *p* < 0.0005, *p* < 0.00005). However, IL-8, IL-10, and TGF-*β* were significantly upregulated compared to the control untreated group (*p* < 0.00005). For IL-12p40, no change in the gene expression was detected. Similarly, in the free drug treatment group, the data demonstrate a downregulation of the expression of IL-1*β* and IL-6 (*p* < 0.000005, *p* < 0.005) and an upregulation of the expression of IL-8 (*p* < 0.00005), IL-10 (*p* < 0.05), TGF-*β* (*p* < 0.05), and TNF-*α* (*p* < 0.00005) genes. No change was noticed for the expression of IL-12p40. In the GNR group, both IL-6 and TNF-*α* showed a significant downregulation in the expression of these genes (*p* < 0.005, *p* < 0.00005), while a significant upregulation of IL-12p40 was detected (*p* < 0.00005). We demonstrate no changes in gene expression or for the remaining cytokines investigated: IL-1*β*, IL-8, IL-10, and TGF-*β* in the GNR group compared to the untreated group (Figure 3).

#### 3.3.2. Protein Secretion Level

For our nanocomplex, the following proinflammatory cytokines: IL-1*β*, IL-6, TNF-*α*, and IL-12p70 were all decreased in a significant manner (*p* < 0.00005, *p* < 0.005, *p* < 0.05, *p* < 0.00005), respectively. While for IL-10, a significant upregulation of the expression level of cells treated with our nanocomplex was detected compared to the control untreated group (*p* < 0.005). On the other hand, no significant change was observed in the secretion level of these two cytokines: IL-8 and TGF-*β*.

**Figure 4:**
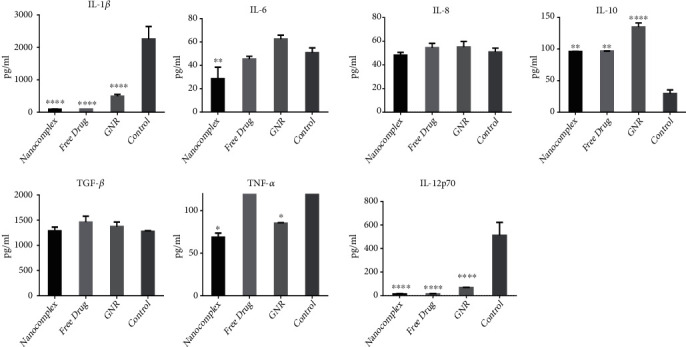
Measurement of the expression level of cytokines secreted by macrophages treated with the following: nanocomplex (a drug-conjugated gold nanorods), the free drug (phosphatidylinositol 3-kinase (PI3K*α*) inhibitor) and GNR; cholesterol-coated gold nanorods and compared to the control untreated cells by using CBA array beads by flow cytometry.

Similarly, for the free drug treatment group, the protein levels in culture for IL-1*β* and IL-12p70 were significantly decreased compared to the control group (*p* < 0.00005, *p* < 0.00005), while an upregulation of the expression level of IL-10 was detected in a statistically significant manner (*p* < 0.005). There are no changes in the levels of IL-6, IL-8, TNF-*α*, and TGF-*β*; on the other hand, for the GNR treatment group, the measured concentrations of IL-1*β*, TNF-*α*, and IL-12p70, for this treatment group, were significantly decreased (*p* < 0.00005,*p* < 0.05,*p* < 0.00005), respectively, while we demonstrate no changes in protein levels for the remaining cytokines investigated; IL-6, IL-8, and TGF-*β* (Figure 4).

## 4. Discussion

Recently, light was shed on the progress of nanotechnology and its rapid spread and development, as manufactured and engineered nanoparticles have been highly involved in the therapeutic industry. However, the clinical use of these particles is still under investigation, as the entrance of these particles into the human body and their interaction with cells is still not clear. Herein, we aimed to investigate the innate immunological response that will result in vitro after using the nanocomplex (a drug-conjugated gold nanorods), the free drug (phosphatidylinositol 3-kinase (PI3K*α*) inhibitor), and the nanocarrier (cholesterol-coated gold nanorods, GNR) compared to the control untreated group. Studying the aforementioned interaction between immune system first responders (macrophages) and these compounds is a step on the right path of assessing the clinical outcomes of using nanocarrier complexed drugs in therapy.

Modulation and alteration in immune receptor expression on the cell surface resulted in an improper response to the intercellular communication processes. Macrophages are known for their diversification, plasticity, and ability to change their phenotype as a response to their local environment which will activate different signaling pathways and induce apoptosis and necrosis cell death modality ([20, 21]; Y. [18]). Interestingly, our nanocomplex acts to induce the polarization of macrophages into a proinflammatory M1-like phenotype, compared to the free drug and GNR, bearing in mind that GNR (gold nanorods) has microbicidal, tumoricidal, and inflammatory activities ([22]; N. [23]).

It is well known that stimulation of macrophages with pathogen-associated molecular patterns (PAMPS) such as LPS drives the differentiation of macrophages to the stated inflammatory phenotype M1 [24]. Here, we investigate the effects on macrophage gene expression and cytokine production upon introduction of these drugs along with LPS. As generally expected, stimulation of macrophages with LPS results in an M1 phenotype which produces and releases higher levels of proinflammatory cytokines, which are potent signaling molecules that are responsible for mediating the intracellular signaling of immune cells, specifically activation of macrophages to produce an effective immune response when exposed to a stimulus [25]. A set of proinflammatory cytokines: TNF-*α*, IL-1*α*, IL-1*β*, IL-6, IL-12, and IL-23 [26] was assessed in the different treatment groups. Additionally, IL-8 is a chemoattractant for immune cells, and it has a key role in activating an immunological response [27]. The regulation, either activation or suppression, of inflammatory response is highly controlled by expressing different antagonist signals, such as the expression of IL-10 and TGF-*β*, which are anti-inflammatory cytokines, that play a major role in suppression the activation of macrophages, by reducing the expression level of the following proinflammatory cytokines: TNF, IL-6, and IL-1 [28, 29]. Our results showed a significant downregulation in the expression level of IL-1*β*, IL-6, and TNF-*α* and a significant upregulation of IL-8, IL-10, and TGF-*β* in the nanocomplex treatment group. While at protein level, the secretion levels of IL-1*β*, IL-6, TNF-*α*, and IL-12p70 were all decreased in a significant manner. And IL-10 was significantly upregulated in cells treated with our nanocomplex, compared to the control untreated group. For the free drug treatment group, the data demonstrate a downregulation of the expression of IL-1*β* and IL-6 and an upregulation of the expression of IL-8, IL-10, TGF-*β*, and TNF- *α* genes, whereas at protein levels, IL-1*β* and IL-12p70 were significantly decreased, and IL-10 was significantly upregulated compared to the control group. In the GNR group, both IL-6 and TNF-*α* showed a significant downregulation in the expression of these genes and a significant upregulation of IL-12p40. On the other hand, at protein levels: IL-1*β*, TNF-*α*, and IL-12p70, this treatment group was significantly decreased only.

It is well known that the gene expression and the protein concentration are not always a perfect correlation, as many regulation steps take place after the transcription of the gene, and make the conclusion in many settings difficult to formulate [30]. Moreover, the differences in the composition of these treatments, nanocomplex (a drug-conjugated gold nanorods), the free drug (phosphatidylinositol 3-kinase (PI3K*α*) inhibitor), and GNR: cholesterol-coated gold nanorods, show different gene regulation and protein production responses. The investigated cytokines have sometimes contradicted or intertwined actions as being regulated and released in response to an inflammation or disease setting. Also, cytokines are well known for being pleotropic, with multiple biological properties. Some have systemic effects while others have microenvironmental effects depending on receptor expression, downstream effects, and the juxtacrine properties of some cytokines [25, 31].

As a result, macrophage polarization is complex and is not restricted to the M1/M2 paradigm [24]. That being said, there are many signaling pathways that can be involved in the polarization of both M1 and M2, and these are responsible to control the switch from one type to the other, making the polarization and activation step not very well understood (N. [23]). The polarization process is highly dynamic and changeable, that it can be reversed and changed from one into another in response to the variation in physiological and pathological conditions [32, 33].

Hence, what we see from our preliminary data on how our nanocomplex: cholesterol-coated drug that is combined with a nanocarrier (Gold nanorods) has different effects on LPS-stimulated macrophages, compared to the free drug (cholesterol) and its sole nanocarrier GNR. Therefore, this is a first step to understand how these drugs work targeting cytokine production by macrophages in response to pathogen invasion.

## 5. Conclusion

We conclude that our nanocomplex seems to be driving the polarization of the derived macrophages into a proinflammatory M1-like phenotype, in which nanocomplex works as a potent effector that can prevent tumoral progression, by switching the surface expression profile of the activated macrophages. Moreover, for cytokine production, our data showed that our nanocomplex has a role in the reduction of proinflammatory cytokines' secretion, in addition to its role in stimulating the expression of anti-inflammatory cytokines IL-10 and TGF-*β*. Additionally, our data points out a strong link between these treatments and increased secretion of the anti-inflammatory cytokine IL-10 by macrophages.

It is worth further investigating and classifying the types of polarized macrophages, taking into account different microenvironments and consecutive cell types that might be involved and activated in disease settings.

## Figures and Tables

**Table 1:** Primer set of inflammatory cytokines.

Gene	F	R
IL-10	GCCAAGCCTTGTCTGAGATGATCC	CATTCTTCACCTGCTCCACGGCC
IL-1*β*	CAGAAGTACCTGAGCTCGCC	AGATTCGTAGCTGGATGCCG
TGF-*β*	GCGCGAGATCCTCTCCATTT	AGGTCCAGCATGAACATGGG
IL12-p40	CATCTGCCTCTTCTTGTGGGT	GACTGGGTCCGAGGGATCTT
TNF	CATCTGCCTCTTCTTGTGGGT	GACTGGGTCCGAGGGATCTT
PPIA “Cyclophilin A”	TCCTGGCATCTTGTCCATG	CCATCCAACCACTCAGTCTTG
IL-6	GGCACTGGCAGAAAACAACC	GCAAGTCTCCTCATTGAATCC
IL-8	CTGGCCGTGGCTCTCTTG	CCTTGGCAAAACTGCACCTT

## Data Availability

Data used to support the findings of this study are included within the article.
